# The effects of Sanxiao Jiuzhi Gong on blood glucose levels and exercise self-efficacy in type 2 diabetes patients: A randomized controlled trial

**DOI:** 10.1097/MD.0000000000044572

**Published:** 2025-09-19

**Authors:** Xia Zhang, Jie-Jie Ge, Yue-Yuan Li, Da-Feng Liu, Bi-Ying Zhang, Xia Zhao, Li Fu, Shi-Ping Feng, Chun-Tao Wu, Shu-Ping Zeng, Bin Wan, Si-Jing Peng

**Affiliations:** a Public Health Clinical Center of Chengdu, Chengdu City, Sichuan, China; b School of Nursing, Anhui University of Chinese Medicine, Hefei City, China; c Key Laboratory of Geriatric Nursing and Health, Anhui University of Chinese Medicine, Hefei City, China.

**Keywords:** blood glucose, exercise self-efficacy, randomized controlled trial, Sanxiao Jiuzhi Gong, type 2 diabetes

## Abstract

**Background:**

Type 2 diabetes mellitus (T2DM) is associated with poor glycemic control and reduced exercise adherence, often influenced by low exercise self-efficacy (ESE). Traditional Chinese Medicine (TCM) exercises, such as Sanxiao Jiuzhi Gong (SJG), may improve both physiological and psychological outcomes in T2DM patients. This study aimed to evaluate the effects of SJG on blood glucose levels and ESE when added to standard diabetes care.

**Methods:**

We conducted a randomized controlled trial involving 60 patients with type 2 diabetes, randomly assigned to either the control group (n = 30) or the SJG group (n = 30). The control group received the standard care for diabetes management, including individualized dietary recommendations, psychological support and aerobic exercise. The experimental group, on the other hand, received the same routine care but also underwent a 12-week intervention of the “ Sanxiao Jiuzhi Gong “ program (3 times/wk, 45 minutes each time). The primary outcome was change in glycated hemoglobin A1c. Secondary outcomes included fasting blood glucose, 2-hour postprandial blood glucose, and ESE scores. All outcomes were assessed at baseline and after the 12-week intervention.

**Results:**

At baseline, no statistically significant differences existed in blood glucose parameters or ESE scores between groups. After 12 weeks, the SJG group demonstrated significantly greater improvements in all glycemic parameters compared to the control group: fasting blood glucose decreased by 3.28 mmol/L versus 1.18 mmol/L (mean difference: 2.10 mmol/L, 95% CI: 0.35–3.84, *P* < .05); 2-hour postprandial blood glucose decreased by 6.05 mmol/L versus 2.03 mmol/L (mean difference: 4.01 mmol/L, 95% CI: 1.38–6.63, *P* < .001); and hemoglobin A1c decreased by 2.73% versus 1.53% (mean difference: 1.20%, 95% CI: 0.20–2.19, *P* < .05). ESE scores increased significantly in the SJG group (2.54 points, *P* < .001) but not in the control group (0.68 points, *P* = .127).

**Conclusion:**

This study provides evidence that SJG exercise, when added to standard care, significantly improves glycemic control and ESE in patients with type 2 diabetes. Future larger-scale, multicenter trials with longer follow-up periods are warranted to confirm these findings and explore the long-term benefits of SJG.

## 1. Introduction

Diabetes mellitus is a chronic metabolic disorder characterized by persistent hyperglycemia resulting from defects in insulin secretion, insulin action, or both.^[1]^. The global prevalence of diabetes mellitus continues to rise, with type 2 diabetes mellitus (T2DM) accounting for approximately 90% to 95% of all cases.^[2]^ Uncontrolled hyperglycemia can lead to severe macrovascular complications (cardiovascular disease, stroke) and microvascular complications (nephropathy, retinopathy, neuropathy), significantly increasing morbidity and mortality.^[3]^

Current evidence-based management of T2DM comprises pharmacotherapy, dietary modification, physical activity, self-monitoring of blood glucose, and diabetes education.^[4]^ Among these interventions, regular physical activity plays a crucial role by improving insulin sensitivity, enhancing glucose utilization, and promoting weight management.^[5]^ However, patient adherence to exercise regimens remains challenging, with factors such as low exercise self-efficacy (ESE) contributing significantly to poor compliance.^[6]^

Traditional Chinese medicine (TCM) has a long history of treating diabetes, referred to as “Xiaoke” (literally “wasting and thirst”). According to TCM theory, T2DM is characterized by 3 primary manifestations: upper “Xiao” (excessive thirst), middle “Xiao” (excessive hunger), and lower “Xiao” (polyuria).^[7]^ The underlying pathophysiology from a TCM perspective involves Yin deficiency and dry heat affecting the lung, stomach, and kidney systems.^[8]^

Recent years have witnessed growing interest in mind-body exercises derived from TCM traditions, such as Tai Chi and Baduanjin, for T2DM management.^[9]^ These practices combine physical movements, breathing techniques, and meditative elements to create comprehensive therapeutic approaches. Systematic reviews and meta-analyses have demonstrated their efficacy in improving glycemic control, reducing insulin resistance, and enhancing quality of life in T2DM patients.^[10,11]^

Sanxiao Jiuzhi Gong (SJG) is based on the theory of Zang-Fu meridians and Yin and Yang 5 elements in TCM, combined with modern medical knowledge of diabetes, emphasizing “combination of Qi and medicine, symptomatic exercise.” Its core principle is to regulate the circulation of Qi and blood, balance Yin and Yang, improve zang-fu functions, especially for Yin deficiency and dryness of lung, stomach and kidney.^[12]^ SJG includes 9 postures (Swallowing saliva; Raising of head; Free from shackles; Soaring to the clouds; Guiding up and down; Hammering to nourish the kidneys; Relieving thirst with Rangu point acupuncture; Clearing obstacles with a hammer; Acupuncture of the Weiyu point), combined with rhythmic breathing and soothing music, stimulates acupoints and meridians through the concept of upper, middle and lower “Xiao” to promote lung health, spleen invigorating and kidney nourishing.^[13]^ However, there is a paucity of randomized controlled studies evaluating the improvement of blood glucose with SJG. Furthermore, the majority of studies have concentrated on the discrepancies in the effectiveness of the exercise intervention modalities, with the potential influence of psychological factors, such as ESE (a pivotal predictor of long-term exercise adherence), remaining unexamined.

ESE is defined as an individual’s confidence in their ability to perform routine physical activities.^[14]^ The promotion of self-efficacy has been demonstrated to be an effective strategy for enhancing patients’ exercise knowledge, thereby facilitating their adherence to regular exercise regimens.^[15]^ Therefore, it is necessary to understand the effects of SJG on ESE in diabetic patients. Therefore, this study aimed to investigate the effects of SJG on both glycemic parameters and ESE in patients with T2DM. We hypothesized that adding SJG to standard diabetes care would result in greater improvements in glycemic control and ESE compared to standard care alone. The findings would provide evidence-based guidance for integrating this TCM exercise approach into comprehensive T2DM management.

## 2. Materials and methods

### 2.1. Study design

We conducted a parallel-group, randomized controlled trial at the Internal Medicine Department of Chengdu Public Health Clinical Medical Center between June 2020 and June 2021. The study protocol was approved by the Medical Ethics Committee of the Chengdu Public Health Center (Approval ID: PJ-K2020-41-01) and registered with the Chinese Clinical Trial Center (Registration Number: ChiCTR2400084254). All participants provided written informed consent before enrollment.

### 2.2. Participants

#### 2.2.1. Eligibility criteria

Patients were eligible if they: met the diagnostic criteria for T2DM according to the Chinese Type 2 Diabetes Prevention and Treatment Guidelines (2020 edition)^[16]^; were aged ≥18 years; had been diagnosed with T2DM for at least 1 year; had not experienced significant traumatic life events in the past month; and voluntarily consented to participate.

Exclusion criteria were: significant blood glucose fluctuations with hypoglycemic tendency; severe liver, kidney, or heart disease; severe diabetes complications or metabolic syndrome; history of mental illness, personality disorders, cognitive impairment, structural brain disorders; or musculoskeletal conditions that would preclude exercise participation.

#### 2.2.2. Sample size calculation

In this study, the sample size was calculated based on the glycated hemoglobin as the primary outcome indicator.^[17]^ Using the PASS 15 software, considering the average difference in glycated hemoglobin, it was determined that each group would have 17 samples. Considering the expected dropout rate of 20%, each group needed at least 20 samples. To enhance the robustness and generalizability of the research results, and taking into account possible dropout or decreased compliance, during the design stage, the ethical review materials and clinical trial registration platform of the study pre-set the maximum sample size for each group at 30, as the reasonable upper limit for expansion.

#### 2.2.3. Randomization and allocation

Eligible participants were randomly assigned to either the SJG group or the control group using a computer-generated random number sequence with a 1:1 allocation ratio. Allocation concealment was maintained using sequentially numbered, opaque, sealed envelopes prepared by a research assistant not involved in participant recruitment or assessment. Due to the nature of the exercise intervention, blinding of participants and intervention providers was not feasible. However, outcome assessors and data analysts were blinded to group allocation.

### 2.3. Interventions

#### 2.3.1. Control group

Participants in the control group received standard diabetes care according to current clinical guidelines, including:

The control group received the standard care for diabetes management, including individualized dietary recommendations, such as clear and controlled diets for gastric heat, fluid-generating diets for lung heat and fluid damage, and Yin and kidney-nourishing diets for Yin deficiency or kidney Yin deficiency. Patients also received emotional support through active listening, guidance on blood glucose monitoring, and diabetes education. Family members were encouraged to provide psychological support. Physical activity recommendations: General advice to engage in moderate-intensity aerobic activity for at least 150 minutes per week.

#### 2.3.2. SJG group

Participants in the SJG group received the standard care described above plus the SJG exercise intervention. The SJG program consisted of 3 45-minute sessions/wk for 12 consecutive weeks, conducted in small groups (5–8 participants) under the guidance of certified SJG instructors. Each session comprised:

Warm-up (10 minutes): Gentle stretching and joint mobilization exercises.
Core SJG exercise (24 minutes): Nine specific movements performed in sequence, each targeting different meridians and acupuncture points relevant to diabetes management:
  Swallowing saliva.
  Raising of head.
  Free from shackles.
  Soaring to the clouds.
  Guiding up and down.
  Hammering to nourish the kidneys.
  Relieving thirst with Rangu point acupuncture.
  Clearing obstacles with a hammer.
  Acupuncture of the Weiyu point.
Cool-down (10 minutes): Relaxation exercises and guided breathing.

The entire exercise sequence was accompanied by specially composed music to facilitate rhythm and promote relaxation. All movements were performed at moderate intensity (40–60% of maximum heart rate), with modifications available for participants with physical limitations.

#### 2.3.3. Intervention fidelity

To ensure standardized delivery of the SJG intervention:

All SJG instructors completed a certified 40-hour training program and passed a competency assessment.
Participants received comprehensive training materials, including printed instructions and demonstration videos.
Weekly supervision sessions were conducted to maintain proper technique.
Attendance was recorded at each session, with follow-up calls for missed sessions.
Random sessions were video-recorded and reviewed by senior instructors for quality control.

### 2.4. Outcome measures

All assessments were conducted at baseline (week 0) and post-intervention (week 12) by trained researchers blinded to group allocation.

#### 2.4.1. Primary outcome

Glycated hemoglobin A1c (HbA1c): Measured using high-performance liquid chromatography (Bio-Rad D-10 HbA1c Program). HbA1c reflects average blood glucose levels over the preceding 2 to 3 months and is the gold standard for monitoring glycemic control.

#### 2.4.2. Secondary outcomes

Fasting blood glucose (FBG): Measured after an overnight fast (≥8 hours) using the glucose oxidase method.

2-hour postprandial blood glucose (2hPG): Measured 2 hours after a standardized meal.

Exercise self-efficacy (ESE): Assessed using the ESE scale, which consists of 9 items rated on a scale from 0 (no confidence) to 10 (complete confidence). The final score is calculated as the mean of all items, with higher scores indicating greater ESE. The Chinese version of ESE scale has demonstrated good reliability (Cronbach’s α = 0.910) and test-retest reliability (0.927) in Chinese populations,^[18]^including those with chronic conditions such as diabetes.^[19]^

#### 2.4.3. Demographic and clinical data

Baseline demographic information collected included age, gender, educational level, marital status, and duration of diabetes. Clinical data included height, weight, body mass index, medication use, and presence of comorbidities.

### 2.5. Statistical analysis

Data analysis was performed using IBM SPSS 25 (IBM Inc., Armonk) . Descriptive statistics, including frequency and percentage, were used to summarize categorical data. Between-group comparisons were conducted using the chi-squared test for categorical data and the Mann–Whitney *U* test for ordinal data. For normally distributed quantitative data, such as FBG, PBG, HbA1c, and ESE, means and standard deviations were reported. Within-group comparisons between pre- and post-intervention data at 12 weeks were performed using paired-samples *t*-tests, and between-group comparisons at the same time points were conducted using independent-samples *t*-tests. Age and duration of diabetes, which did not follow a normal distribution, were presented as medians, and between-group comparisons were conducted using the Mann–Whitney *U* test. A significance level of *P* < .05 was considered statistically significant.

## 3. Results

### 3.1. Participant flow and characteristics

**Figure 1.**
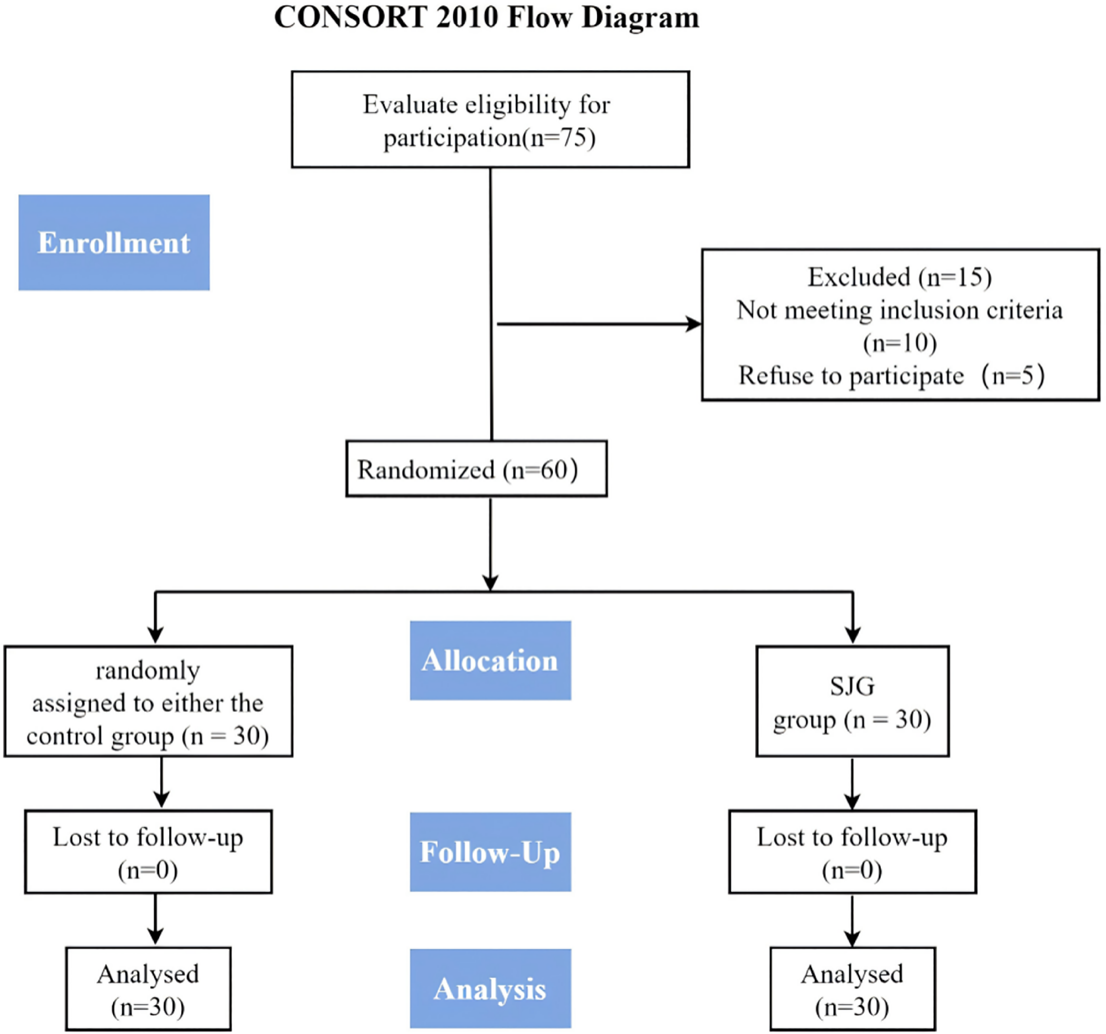
CONSORT flow diagram. SJG = Sanxiao Jiuzhi Gong.

Figure 1 presents the CONSORT flow diagram of participant progress through the trial. Of 75 patients screened, 60 met the eligibility criteria and were randomized to either the SJG group (n = 30) or the control group (n = 30). All participants completed the 12-week study period, resulting in a 0% attrition rate. This exceptional retention was attributed to the comprehensive follow-up protocol, which included weekly telephone check-ins, flexible scheduling of sessions, and involvement of family members in supporting participation.

Table 1 presents the baseline demographic and clinical characteristics of participants. The sample was predominantly male (SJG group: 83.3% male; control group: 86.7% male), reflecting the gender distribution of T2DM patients in our hospital during the recruitment period. The median age was 54.50 years in the SJG group and 55.50 years in the control group. No statistically significant differences were observed between groups in any baseline characteristic (all *P* > .05), indicating successful randomization.

**Table 1:** Demographic baseline information comparison.

		Control group (n = 30)	SJG group (n = 30)	χ^2^ and *Z* value	*P* value
Gender	Male	26 (86.67%)	25 (83.33%)	χ^2^ = 0.131	.718
	Female	4 (13.33%)	5 (16.67%)		
Age	18	10 (33.33%)	13 (43.33%)	*Z* = 0.635	.426
	≥50	20 (66.67%)	17 (56.67%)		
Education	Without education	5 (16.67%)	1 (3.33%)	χ^2^ = 6.003	.199
	Elementary school	4 (13.33%)	2 (6.67%)		
	Middle school	9 (30.00%)	9 (30.00%)		
	High school	6 (20.00%)	13 (43.33%)		
	College or above	6 (20.00%)	5 (16.67%)		
Marital status	Unmarried	4 (13.33%)	3 (10.00%)	χ^2^ = 0.476	.924
	Married	24 (80.00%)	24 (80.00%)		
	Divorced	1 (3.33%)	2 (6.67%)		
	Widow/widower	1 (3.33%)	1 (3.33%)		
History of DM	0–5	14 (46.67%)	18 (60.00%)	Z = 0.735	.391
	5–10	6 (20.00%)	4 (13.33%)		
	10–15	7 (23.33%)	5 (16.67%)		
	≥15	3 (10.00%)	3 (10.00%)		

DM = diabetes mellitus, SJG = Sanxiao Jiuzhi Gong.

### 3.2. Blood glucose level

The changes in glycemic parameters from baseline to week 12 for both groups. At baseline, there were no significant differences between groups in FBG, 2hPG, or HbA1c (all *P* > .05).

After 12 weeks, both groups showed significant improvements in all glycemic parameters compared to baseline (all *P* < .05). However, the SJG group demonstrated significantly greater improvements than the control group in FBG (mean difference: 2.10 mmol/L, 95% CI: 0.35–3.84, *P* < .05), 2hPG (mean difference: 4.01 mmol/L, 95% CI: 1.38–6.63, *P* < .001), and HbA1c (mean difference: 1.20, 95% CI: 0.20–2.19, *P* < .05).The results are shown in Tables 2 and 3.

**Table 2:** Comparison of blood glucose levels.

	Patient number	FPG (mmol/L)		2hPG (mmol/L)		HbA1c (%)	
		Baseline	12 wk post-intervention	Baseline	12 wk post-intervention	Baseline	12 wk post-intervention
Control group	30	8.43 ± 3.73	7.25 ± 2.10	12.31 ± 4.70	10.27 ± 3.98	8.85 ± 1.57	7.32 ± 1.98
SJG group	30	9.57 ± 3.48	6.29 ± 1.16	14.27 ± 5.23	8.22 ± 2.38	8.98 ± 2.33	6.25 ± 1.01
*t* value		1.218	−2.187	1.525	−2.424	0.253	−2.630
*P* value		.228	.033	.133	.019	.801	.011

2hPG = 2-hour postprandial blood glucose, FBG = fasting blood glucose, HbA1c = hemoglobin A1c, SJG = Sanxiao Jiuzhi Gong.

**Table 3:** Comparison of FBG, 2hPG and HbA1c levels.

Groups	Patient number	FBG (mmol/L)	2hPG (mmol/L)	HbA1c (%)
The control group	30	1.18	2.03	1.53
The SJG group	30	3.28	6.05	2.73
*t* value		−2.187	0.133	−2.630
*P* value		.033	.019	.011

2hPG = 2-hour postprandial blood glucose, FBG = fasting blood glucose, HbA1c = hemoglobin A1c, SJG= = Sanxiao Jiuzhi Gong.

### 3.3. Exercise self-efficacy outcomes

At baseline, there were no statistically significant differences in ESE scores between the 2 groups (*P* > .05).

After 12 weeks of intervention, patients in the SJG group showed a significant increase of 2.54 points in their ESE scores compared to baseline (mean difference: 2.54, 95% CI: 1.63–3.56 *P* < .001). However, in the control group, there was no statistically significant difference in ESE scores between the 12-week intervention and baseline (*P* > .05). Data are shown in Table 4.

**Table 4:** The ESE score comparison.

Groups	Patient number	Baseline ESE score	ESE scores post-intervention	Difference of ESE scores	*t* value	*P* value
The control group	30	3.71 ± 1.99	4.38 ± 2.61	0.67	−1.571	.127
The SJG group	30	4.01 ± 1.83	6.55 ± 1.88	2.54	−5.693	.000
*t* value		0.616	3.691			
*P* value		.541	.000			

ESE = exercise self-efficacy, SJG = Sanxiao Jiuzhi Gong.

## 4. Discussion

This randomized controlled trial examined the effects of a 12-week SJG exercise intervention on glycemic control and ESE in patients with T2DM. Our findings demonstrate that adding SJG to standard diabetes care resulted in significantly greater improvements in glycemic parameters (FBG, 2hPG, HbA1c) and ESE compared to standard care alone.

### 4.1. Effects on glycemic control

The significant improvements in glycemic parameters observed in the SJG group align with previous studies of TCM exercise interventions for diabetes management. A meta-analysis by Wang et al found that traditional Chinese exercises significantly reduced HbA1c (weighted mean difference: −0.77%, 95% CI: −1.15% to −0.39%) in patients with T2DM.^[11]^ Our study demonstrated an even larger reduction in HbA1c (−2.73%) with SJG, suggesting that this diabetes-specific exercise system may offer enhanced benefits compared to more general TCM exercises.

SJG in the “guide up to lead down” action, the upper take Yunmen, the lower take kidney Yu. TCM believes that Yunmen, in addition to helping to prevent and control cough, asthma, chest tightness and other symptoms, to clear the chest of the annoying heat also has a significant effect, so by the lung heat injuries to the fluid, the annoying heat and drinking “on the elimination of” has a certain role. Secondly, Chinese medicine believes that the thirst syndrome in the consumption due to spleen weakness, the function of the kidney is the main hidden essence, the main water, regulating the body’s metabolism of the whole body water, so enhance the human body’s kidney function can help to improve the thirst syndrome under the symptoms of thirst.^[20]^ In addition, the human body there are twelve main meridians and odd meridians and veins, the former is belongs to the 5 viscera and 6 bowels, the meridians and is the human body qi and blood running channel, and with the internal organs, the human body meridians and veins smooth qi and blood smooth, thus increasing the spleen, lungs and kidneys, so that the functions of the organs and bowels of the harmonious function.^[21]^ Chinese medicine SJG can be taken through the meridians and take the meridians to click, knocking points, to promote the patient meridian smooth, so as to improve the patient’s blood glucose, blood lipid levels.

Insulin resistance and pancreatic β-cell dysfunction are 2 key pathophysiologic mechanisms in the development of type 2 diabetes.^[22]^ SJG is guided by the theory of Chinese medicine for the treatment of thirst, through the synergistic effect of form and spirit co-nourishment (body regulation), emotional and emotional guidance (heart regulation), and respiratory regulation (breath regulation), together with the meridian guidance and exercise at specific meridian points, thus it may systematically enhance insulin sensitivity, and also improve the glucose and lipid metabolism homeostasis by regulating the function of autonomic nerves, and its low-intensity, sustained exercise characteristics are in line with the safe and effective aerobic exercise standards recommended by the WHO.

### 4.2. Effects on exercise self-efficacy

ESE refers to an individual’s confidence in their ability to plan and execute physical activities when faced with various challenging situations.^[18]^ Ke Sanmei and colleagues have shown that aerobic exercise can effectively enhance the ESE levels among hemodialysis patients. This improvement is translated to increased confidence in maintaining exercise routines and reducing negative emotions and fatigue.^[23]^ Consistent with these findings, our study revealed that following a 12-week intervention, participants in the SJG group exhibited a substantial 2.54-point increase in their ESE scores, while the control group showed a more modest 0.67-point increase.

Our findings can be attributed to the pivotal roles of cognition, behavior, and emotions in shaping the ESE. In our study, patients received comprehensive training that imparted essential knowledge and skills related to the SJG exercise program. This training fortified their cognitive and behavioral processes associated with exercise and fostered a positive state of mind. This, in turn, guided patients towards adopting constructive self-guidance in their behaviors and emotions. Consequently, this approach aided in the regulation of negative emotions such as anxiety and depression, resulting in a dual positive impact on both psychological and physiological aspects. Ultimately, this contributed to strengthen patients’ belief in their ability to perform these exercises.

Additionally, there exists a negative correlation between blood glucose levels and ESE, indicating that patients with higher ESE scores tend to have lower blood glucose levels.^[21]^ In this study, the application of the SJG training to type 2 diabetes patients improved blood glucose control by adjusting Qi and blood, strengthening the lungs, invigorating the spleen, and nourishing the kidneys. This enhancement of ESE improved the self-management capabilities of diabetes patients, which had a positive effect on their recovery, disease stability, and skeletal muscle condition.

### 4.3. Limitations

There are some limitations to this study. First, a high percentage of subjects in this study were male (83–87%), resulting in an uneven gender distribution. This gender imbalance may affect the generalizability of the study results, especially among women with type 2 diabetes. Previous studies have shown that there may be differences between men and women in the pathogenesis of diabetes, treatment response, and self-management behaviors. Therefore, the results of the current study may not be fully representative in the female population. It is recommended that future studies focus on gender balance when recruiting subjects to improve the external validity of the findings. In addition, a notable limitation of the current study was the selection of the control group. Participants in the control group received an active intervention, which may have diluted the amount of observed effect of the SJG intervention. The use of an active control made it difficult to separate the specific effects of SJG from the general treatment effects. Future studies should consider using a sham exercise group or wait-list control to better distinguish the unique contributions of SJG. Finally, this study was short and conducted in only 1 hospital, and subsequent studies could focus on extending the post-intervention follow-up observation period, conducting multicenter randomized controlled trials, and so on, in order to scientifically prove the long-term intervention effect of SJG training.

### 4.4. Clinical implications

Our findings suggest that SJG could be a valuable addition to standard diabetes care, particularly for patients who may benefit from a gentle, culturally appropriate exercise option. The improvements in both glycemic control and ESE suggest that SJG addresses both physiological and psychological aspects of diabetes management.

Healthcare providers should consider referring patients with T2DM to SJG programs, especially those who have not responded adequately to conventional exercise recommendations or who express interest in mind-body approaches. The exercise is low-impact, requires no special equipment, and can be adapted for individuals with physical limitations, making it accessible to a wide range of patients.

From a public health perspective, SJG could be implemented in community settings as a cost-effective group intervention. The format is conducive to peer support and social interaction, which may enhance engagement and adherence.

## 5. Conclusion

This randomized controlled trial provides evidence that a 12-week Sanxiao Jiuzhi Gong exercise intervention, when added to standard care, significantly improves glycemic control and ESE in patients with type 2 diabetes. The magnitude of improvements in HbA1c and other glycemic parameters suggests that SJG may offer clinically meaningful benefits for diabetes management. Future research should address the limitations of the current study by conducting larger multi-center trials with longer follow-up periods, more balanced gender representation, and direct assessment of physiological mechanisms. Studies comparing SJG with other established exercise interventions for T2DM would also help clarify its relative efficacy and unique benefits.

Additionally, qualitative research exploring participants’ experiences with SJG could provide valuable insights into factors affecting engagement and adherence. Investigation of SJG’s potential effects on diabetes complications, mental health outcomes, and overall quality of life would further enhance our understanding of its comprehensive benefits.

In conclusion, SJG represents a promising complementary approach to T2DM management that merges traditional wisdom with contemporary healthcare needs. By simultaneously addressing physiological parameters and psychological factors like ESE, SJG embodies an integrative approach that may help bridge the gap between treatment recommendations and sustained patient engagement in self-care behaviors.

## Acknowledgements

The authors thank all healthcare professionals and expert panel members who contributed their time and expertise to this study. We also acknowledge TopEdit (www.topeditsci.com) for providing linguistic assistance during manuscript preparation.

## Author contributions

**Conceptualization:** Xia Zhang, Jie-Jie Ge, Xia Zhao, Chun-Tao Wu, Si-Jing Peng.

**Data curation:** Yue-Yuan Li, Da-Feng Liu, Bi-Ying Zhang, Li Fu, Shi-Ping Feng.

**Formal analysis:** Yue-Yuan Li, Shi-Ping Feng.

**Funding acquisition:** Bin Wan.

**Investigation:** Jie-Jie Ge, Xia Zhao, Bin Wan.

**Methodology:** Da-Feng Liu, Shi-Ping Feng, Chun-Tao Wu.

**Project administration:** Xia Zhao, Chun-Tao Wu, Bin Wan.

**Resources:** Li Fu, Shu-Ping Zeng.

**Software:** Li Fu, Shu-Ping Zeng.

**Supervision:** Si-Jing Peng.

**Validation:** Jie-Jie Ge, Shu-Ping Zeng.

**Writing** – **original draft:** Xia Zhang.

**Writing** – **review & editing:** Yue-Yuan Li, Da-Feng Liu, Bi-Ying Zhang, Li Fu, Shi-Ping Feng, Si-Jing Peng.
